# Cost of anxiety disorders in Japan in 2008: a prevalence-based approach

**DOI:** 10.1186/1471-244X-13-338

**Published:** 2013-12-18

**Authors:** Mitsuhiro Sado, Sayuri Takechi, Ataru Inagaki, Daisuke Fujisawa, Akihiro Koreki, Masaru Mimura, Kimio Yoshimura

**Affiliations:** 1 Department of Neuropsychiatry, Keio University School of Medicine, Shinanomachi 35, Shinjuku-ku, Tokyo 160-8582, Japan; 2 Division of Psychiatry, Seiwa Hospital, Bentencho 9, Shinjuku-ku, Tokyo 162-0851, Japan; 3 Psycho-Oncology Division, National Cancer Center East, Japan, 6-5-1 Kashiwanoha, Kashiwa, Chiba 277-8577, Japan; 4 Department of Health Policy and Management, Keio University School of Medicine, Shinanomachi 35, Shinjuku-ku, Tokyo 160-8582, Japan

**Keywords:** Cost of illness, Anxiety disorders, Societal burden, Cost analysis, Societal costs

## Abstract

**Background:**

The societal burden caused by anxiety disorders has likely been underestimated, while those for schizophrenia and depression have received more attention. Anxiety disorders represent a significant illness category that occurs at a high prevalence and poses a considerable burden. However, the cost of anxiety disorders in Japan has not yet been well researched. The goal of the present study was to estimate the total cost of anxiety disorders in Japan and to clarify the characteristics of this burden.

**Method:**

A prevalence-based approach was adopted to measure the total cost of anxiety disorders. Anxiety disorders were defined as diagnosis code F40.0-F41.9 according to the International Statistical Classification of Diseases and Related Health Problems, 10th Revision. The cost was comprised of the following components: medical treatment costs and social service costs as direct costs, and morbidity and mortality costs as indirect costs. Data were collected from publicly available statistics.

**Results:**

The total cost of anxiety disorders in Japan in 2008 was JPY 2.4 trillion (US$ 20.5 billion at the current exchange rate of US$1 = JPY 116.8). The direct cost was JPY 50 billion. The morbidity cost was JPY 2.1 trillion, while the mortality cost was JPY 0.24 trillion.

**Conclusions:**

The social burden caused by anxiety disorders in Japan is tremendous and is similar to that of other mental disorders. Productivity loss in the workplace represents the largest portion of all the cost components. Because the medical examination rate is quite low, the improvement of healthcare access might contribute to cost mitigation.

## Background

Mental illness has become an issue of serious concern. According to a WHO survey conducted in 2010, an estimated 450 million persons worldwide suffer from mental or behavioral diseases [1], posing a considerable burden on society. In Japan, for example, the accrued cost of depression in 2005 was JPY 2.0 trillion (USD 18 billion) [2], while that of schizophrenia in 2008 was JPY 2.8 trillion (USD 24 billion) [3]. Anxiety disorders also represent a significant illness category that occurs at a high prevalence and that poses a considerable burden. For example, the 12-month prevalence rates of anxiety disorders were estimated to be over 10% in the US and Europe (18.1% in the US [4]; 13.6% in Europe [5]). Although the prevalence of anxiety is estimated to be lower in Japan (5.5%), it remains twice as high as that of depression [6]. As for the societal burden caused by anxiety disorders, the burden was estimated to be within the range of USD 42.3 billion to USD 46.6 billion in the U.S. in 1990 [7,8] and approximately GBP 1.2 billion in England in 2007 [9].

A tendency for indirect costs to constitute the major portion of the total costs was common among these studies. Therefore, a precise estimation of the indirect and direct costs is essential for evaluating the societal cost of this illness category. The cost of anxiety disorders in Japan has not yet been researched adequately.

### Objective

This study attempted to estimate the cost caused by anxiety disorders in Japan in 2008, so as to provide information regarding the extent of the societal burden posed by anxiety disorders.

## Methods

A prevalence-based approach was used to measure the total costs caused by anxiety disorders among adults over 20 years of age in Japan in 2008. Data for 2008 were used, as this was the most recent year for which all the necessary data were available. Anxiety disorders were defined as diagnosis code F40.0-F41.9 according to the International Statistical Classification of Diseases and Related Health Problems, 10th Revision (ICD-10). The total cost was comprised of the following components: healthcare costs and social service costs as direct costs, and morbidity and mortality costs as indirect costs. The informal care cost was excluded because of the lack of a reliable means of estimating this cost in Japan. This study was conducted from a societal perspective. Data were collected from publicly available statistics and reports. We have acknowledged the possibility that data derived from these statistics varied depending on the year surveyed, since the data were estimated using a random sampling method. Therefore, when previous data were available [10,11], the data were checked and the stability of the data was confirmed. Analyses were conducted according to conservative principles (i.e., the avoidance of overestimates). The results are shown in both Japanese yen (JPY) and US dollars (USD). The Purchasing Power Parity between JPY and USD for 2008 (USD 1 = JPY 116.8) was used to calculate the USD equivalent of each cost component.

### Direct costs

#### Healthcare costs covered by health insurance

To calculate the healthcare costs covered by health insurance, we mainly used two Ministry of Health, Labour and Welfare data sources: the Patient Survey [10] and the Survey of Medical Care Activities in Public Health Insurance [11]. In Japan, universal healthcare is provided under public health insurance schemes. The volume of services provided under these schemes is reported annually by the Survey of Medical Care Activities in Public Health Insurance [11], while the number of patients (categorized according to diagnosis) utilizing healthcare services within these schemes is estimated every three years by the Patient Survey [10]. Briefly, the Patient Survey estimated the number of patients in each diagnostic category for all physical and mental diseases, while the Survey of Medical Care Activities in Public Health Insurance showed the treatment expenses covered by public health insurance in each diagnostic category. Regarding anxiety disorders, the Survey of Medical Care Activities in Public Health Insurance showed the total combined outpatient costs for all F4 group disorders, including panic disorders, social anxiety disorders, somatoform disorders, dissociative disorders and so on; thus, the costs specifically associated with anxiety disorders were not indicated. Accordingly, the proportion of patients with anxiety disorders among all the patients with F4 group disorders was calculated so as to examine the costs associated only with anxiety disorders, assuming that the average outpatient cost among patients with different F4 group disorders was the same.

Healthcare costs covered by health insurance included all costs needed to provide each healthcare service, such as salaries for physicians and staffs, medical devices, medications, overhead costs, training costs, and so on.

#### Outpatient cost

Outpatient cost data were collected from the Patient Survey [10] and the Survey of Medical Care Activities in Public Health Insurance [11]. However, the Survey of Medical Care Activities in Public Health Insurance [11] showed only the combined medical costs for F4 group disorders (neurotic, stress-related, and somatoform disorders); thus, the specific costs caused by anxiety disorders could not be estimated. As for the data from the Patient Survey [10], the proportion of patients with anxiety disorders among all the patients diagnosed as having F4 disorders was calculated, and the proportional cost caused by anxiety disorders alone per month was calculated from the total healthcare cost for F4 disorders, assuming that the average outpatient costs for the different F4 disorders were the same. The formula shown below was used to calculate the annual outpatient costs:

$$C_{\mathrm{anx}-\mathrm{out}}=C_{f4-\mathrm{out}}\times \frac{N_{\mathrm{anx}-\mathrm{out}}}{N_{f4-\mathrm{out}}}\times 12,$$

where *C*_*anx-out*_ is the outpatient cost of anxiety disorders, *C*_*f4-out*_ is the outpatient cost of the F4 group, *N*_*anx-out*_ is the estimated number of outpatients with anxiety disorders, and *N*_*f4-out*_ is the estimated number of outpatients with F4 group diagnoses.

#### Inpatient cost

Inpatient cost data were also obtained from the Patient Survey [10] and the Survey of Medical Care Activities in Public Health Insurance [11]. To calculate the proportion of patients with anxiety disorders among all the patients diagnosed as having F4 disorders and the proportional cost for anxiety disorders alone per month, the same method as that used to calculate the outpatient cost was applied. The formula shown below was used to calculate the annual inpatient cost:

$$C_{\mathrm{anx}-\mathrm{in}}=C_{f4-\mathrm{in}}\times \frac{N_{\mathrm{anx}-\mathrm{in}}}{N_{f4-\mathrm{in}}}\times 12,$$

where *C*_*anx-in*_ is the inpatient cost of anxiety disorders, *C*_*f4-in*_ is the inpatient cost of the F4 group, *N*_*anx-in*_ is the estimated number of inpatients with anxiety disorders, and *N*_*f4-in*_ is the estimated number of inpatients with F4 group diagnoses.

#### Medication cost

The medication cost was regarded as the total cost of any medications prescribed for patients with a diagnosis of anxiety disorder. The medication cost data were collected from the Patient Survey [10] and the Survey of Medical Care Activities in Public Health Insurance [11]. Because of the above-mentioned limitation of the Survey of Medical Care Activities in Public Health Insurance [11], the same method (i.e., utilizing the Patient Survey) as that used to calculate the outpatient and inpatient costs was applied. The formulas used to calculate the inpatient and outpatient medication costs are shown below:

$$C_{\mathrm{anx}-\mathrm{in}-m}=C_{f2-\mathrm{in}-m}\times \frac{N_{\mathrm{anx}-\mathrm{in}}}{N_{f2-\mathrm{in}}}\times 12,\mathrm{and}$$

$$C_{\mathrm{anx}-\mathrm{out}-m}=C_{f4-\mathrm{out}-m}\times \frac{N_{\mathrm{anx}-\mathrm{out}}}{N_{f4-\mathrm{out}}}\times 12,$$

where *C*_*anx-in-m*_ and *C*_*anx-out-m*_ are the inpatient and outpatient medication costs of anxiety disorders, *C*_*f4-in-m*_ and *C*_*f4-out-m*_ are the inpatient and outpatient medication costs of the F4 group, *N*_*anx-in*_ and *N*_*anx-out*_ are the estimated numbers of inpatients and outpatients with anxiety disorders, and *N*_*f4-in*_ and *N*_*f4-out*_ are the estimated numbers of inpatients and outpatients with F4 group diagnoses, respectively. Medication costs covered by the “Diagnosis Procedure Combination/Per-diem Payment System” (DPC/PDPS) were excluded from the inpatient medication costs calculated above, as these costs had already been included in the inpatient cost of anxiety disorders calculated in the previous section.

#### Involuntary admission cost

As described by Sado et al. [3], two distinct types of formal admission exist: ‘medical protection admission’ and ‘involuntary admission' . Therefore, a patient can be admitted to a hospital through three pathways in Japan: 1) a voluntary admission, to which the patient consents; 2) a ‘medical protection admission’ , which occurs when a designated psychiatrist acts in compliance with the “Law Related to Mental Health and Welfare of Persons with Mental Disorders” and judges a patient to have a mental disorder requiring inpatient treatment and the patient’s guardian agrees to the admission and treatment, even though the patient himself/herself might not provide consent; and 3) an ‘involuntary admission' , which occurs when the assessments of two independent, designated psychiatrists conclude that a patient has a mental illness and presents a risk to himself/herself or to others.

The Law Related to Mental Health and Welfare of Persons with Mental Disorders, which is similar in some ways to the UK’s Mental Health Act of 1983, is described by the Law Society as follows: The Law Related to Mental Health and Welfare of the Person with Mental Disorder, similar in some ways to the UK’s Mental Health Act 1983, is described by the Law Society thus: The law to provide person(s) with mental disorder with medical care and protection, and in combination with the Law to Support Independence of Disabled Persons to offer necessary assistance for promoting their social rehabilitation, self-support and participation in socio-economic activities, and to endeavor to prevent onset thereof, maintain and promote mental health of the people in general, to thereby advance general well-being of the person(s) with mental disorder and to enhance mental health of the people in general [12].

Under the arrangements for voluntary admissions and ‘medical protection admissions' , the costs are met by health insurers in the usual way; thus, the costs are included in the inpatient admission costs described above. However, ‘involuntary admission’ costs are met by a separate budget that is directly funded by taxpayers and thus needs to be added to the inpatient costs described above.

The involuntary admission cost was estimated as follows. Using data from the Mental Health and Welfare Document [13], we obtained the number of involuntary admissions per day and multiplied this number by the healthcare costs per day; this value was then multiplied again by 365 (the number of days in a year). The medical cost per day for involuntary admission was assumed to be the same as the hospital costs for patients with F4 disorders, as estimated by the Survey of Medical Care Activities in Public Health Insurance [11].

### Costs of provision under the Medical Care and Supervision Act in Japan

The Medical Care and Supervision Act in Japan defines the provision of appropriate medical care and treatment to promote social rehabilitation for persons who have committed serious crimes under the condition of insanity or diminished capability [14]. The budget for the execution of mental health supervision and the execution of medical care provision under the Medical Care and Supervision Act in Japan was used as the starting point to calculate the cost of provision under the Medical Care and Supervision Act in Japan. The rate of patients with anxiety disorders among all the patients treated under the Medical Care and Supervision Act in Japan was not available but was calculated by hypothesizing that the rate of the number of F4 group patients among all the psychiatric patients and the rate of the number of patients with anxiety disorders among all the F4 group patients under the Medical Care and Supervision Act in Japan would be the same as the rate of the number of F4 group patients among all the psychiatric patients admitted involuntarily [13] and the rate of the number of patients with anxiety among all the F4 group patients under the health insurance schemes from the Patient Survey [10], respectively.

#### Social service cost

The social service cost represents the cost of services provided under the Services and Supports for Persons with Disabilities Act. The cost of all the services provided under the Services and Supports for Persons with Disabilities Act was regarded as the total social service costs. As described by the Ministry of Health, Labour and Welfare, this Act aims to improve the welfare of persons (adults) and children with disabilities through the provision of benefits for necessary disability welfare services and the provision of other forms of support to enable persons (adults) and children with disabilities to live independent daily or social lives according to their respective abilities and aptitudes [15]. The costs of other social services provided outside the scope of this act, such as the medical assistance costs covered by public assistance (i.e., financial support for medical treatment costs by local government for those receiving public assistance) and the costs for health centers and group homes, should be included in this cost; however, these components had to be excluded from the analysis because of the lack of available data. The associated data used to calculate this cost were obtained from a Mental Health and Welfare Document [13] and a study titled “Survey of patients with mental disorders attending mental health clinics who are not involved in social activities currently, and research to improve the support of patients wishing to become involved in social activities” conducted in 2007 by the Japanese Association of Mental Health Services (*ASUKURI* research) [16]. The services provided under the Services and Supports for Persons with Disabilities Act include those that provide places and opportunities for employment (type A support) or training without an employment contract (type B support) for patients with disabilities who have difficulty obtaining employment from for-profit organizations. First, we added the number of those who use support for continuous employment (type A) or support for continuous employment (type B). Then, we applied data on the frequency of use to each diagnosis in the *ASUKURI* research [16] and calculated the total number of people using these offices per year, multiplying the total number by the cost per single use.

### Indirect costs

The indirect costs were comprised of the morbidity cost (including absenteeism, presenteeism and unemployment cost) and the mortality cost. Morbidity costs occur when patients are not able to function normally as a result of their illness [17], while mortality costs arise when patients die as a result of suicide at an age earlier than the average life expectancy.

#### Morbidity cost

Morbidity cost represents the loss resulting from a decline in the productivity of employees with anxiety disorders (i.e., absenteeism and presenteeism) and the loss resulting from the lack of employment of patients with anxiety disorders (i.e., unemployment cost).

##### Absenteeism and presenteeism

To estimate absenteeism and presenteeism resulting from anxiety disorders, first we estimated absenteeism arising from each of the disorders that are classified as anxiety disorders (i.e., agoraphobia, panic disorder, social anxiety disorder [SAD], specific phobias, and generalized anxiety disorder [GAD]), by multiplying the number of patients for each sex and age group by the average number of days of suspension from employment and the expected daily earning, then adding up the costs for each sex and age group.

The number of patients was estimated according to sex and age by multiplying the population, according to sex and age, and the 12-month prevalence rate. The 12-month prevalence rate, according to sex and age, and the average number of days of suspension from employment were retrieved from the World Mental Health survey of Japan (WMH-J) report (Tables 1 and 2) [6]. The expected daily earning was calculated based on the Basic Survey on Wage Structure [18] and the Monthly Labour Survey [19] (Table 3). The formula used to calculate the cost of absenteeism for each sex and age group was as follows:

$$C_{\mathrm{ab}}=P_{o}\times P_{r}\times D_{\mathrm{ab}}\times W,$$

where *C*_*ab*_ is the cost of absenteeism, *P*_*o*_ is the population size*, P*_*r*_ is the 12-month prevalence of anxiety disorders, *D*_*ab*_ is the average number of days of suspension from employment, and *W* is the expected average daily earning.

**Table 1:** Prevalence and standard error (SE) of anxiety disorders for age and sex

		**Male**			**Female**		
	**Age**	**Prevalence**	**SE**	**Distribution**	**Prevalence**	**SE**	**Distribution**
Agoraphobia	20-34	0.012	0.006	beta	0.017	0.007	beta
	35-44	0.005	0.004	beta	0.007	0.005	beta
	45-54	0.003	0.003	beta	0.004	0.003	beta
	55-64	0.002	0.002	beta	0.003	0.002	beta
	65-	0.001	0.002	beta	0.002	0.002	beta
SAD	20-34	0.018	0.008	beta	0.014	0.006	beta
	35-44	0.013	0.007	beta	0.010	0.005	beta
	45-54	0.009	0.005	beta	0.007	0.004	beta
	55-64	0.004	0.003	beta	0.003	0.003	beta
	65-	0.004	0.003	beta	0.003	0.002	beta
Specifc Phobia	20-34	0.032	0.010	beta	0.045	0.011	beta
	35-44	0.034	0.011	beta	0.048	0.012	beta
	45-54	0.028	0.009	beta	0.040	0.009	beta
	55-64	0.021	0.007	beta	0.030	0.008	beta
	65-	0.017	0.006	beta	0.024	0.006	beta
Panic Disorder	20-34	0.005	0.004	beta	0.009	0.005	beta
	35-44	0.005	0.004	beta	0.008	0.005	beta
	45-54	0.007	0.004	beta	0.013	0.005	beta
	55-64	0.001	0.001	beta	0.001	0.002	beta
	65-	0.017	0.006	beta	0.024	0.006	beta
GAD	20-34	0.010	0.006	beta	0.016	0.006	beta
	35-44	0.010	0.006	beta	0.016	0.007	beta
	45-54	0.009	0.005	beta	0.014	0.006	beta
	55-64	0.006	0.004	beta	0.010	0.005	beta
	65-	0.003	0.002	beta	0.005	0.003	beta

Calculated by the authors based on the data derived from Kawakami (2006).

**Table 2:** Lost work days for each anxiety disorder and relative ratio of day-equivalents for presenteeism versus absenteeism

**Diagnosis**	**Lost work days because of absenteeism**		**Relative ratio of day-equivalents for presenteeism versus absenteeism**	
	**days (SE)**	**distribution**	**ratio**	**distribution**
Agoraphobia	7.3(6.14)	gamma	3.26	deterministic
SAD	7.3(6.14)	gamma	3.26	deterministic
Specifc Phobia	1.3(0.84)	gamma	3.26	deterministic
Panic	7.3(6.14)	gamma	3.26	deterministic
GAD	8.7(2.88)	gamma	3.26	deterministic

Derived from Kawakami (2006).

**Table 3:** Expected daily earnings

	**male**		**female**	
**Age**	**JPY**	**distribution**	**JPY**	**distribution**
20-24	12,788	deterministic	11,515	deterministic
25-29	16,180	deterministic	13,872	deterministic
30-34	19,411	deterministic	14,796	deterministic
35-39	22,777	deterministic	15,752	deterministic
40-44	25,992	deterministic	16,251	deterministic
45-49	27,686	deterministic	15,625	deterministic
50-54	27,731	deterministic	15,265	deterministic
55-59	25,631	deterministic	14,310	deterministic
60-64	17,484	deterministic	11,746	deterministic
65-69	14,586	deterministic	11,097	deterministic
70-	16,178	deterministic	12,444	deterministic

Derived from Basic Survey on Wage Structure.

On the other hand, we were not able to obtain reliable data for presenteeism in Japan. Therefore, similar to the protocol used in a previous study [2], we decided to conduct a literature review to determine the relative ratio of days of presenteeism versus those of absenteeism. Studies were included in the results of the literature review if they met the following conditions:

•an observational study performed in a large, representative, community sample taken from the general population, and

•the rates of absenteeism and presenteeism were measured directly from the samples, and anxiety disorders was defined using a recent psychiatric diagnostic classification system, such as the ICD, or DSM, to distinguish anxiety from ill-defined psychological distress or stress as an outcome.

We excluded studies using workplace samples because such studies were unlikely to represent a diversity of vocations. The evidence was further restricted to peer-reviewed, published, English language reports. We performed the literature review using PubMed and the following search terms: anxiety disorder, absenteeism, presenteeism, and productivity loss. As a result, only one article [20] met the above-mentioned criteria for inclusion in the search results. Briefly, this study estimated the cost of lost productivity due to a variety of medical conditions, such as allergic rhinitis, anxiety disorders, depression, hypertension, and so on, using data from 8,267 US employees at 47 employer locations. The results showed that the cost of presenteeism associated with anxiety disorders was 3.26 times higher than that of absenteeism.

The number of equivalent days of presenteeism was thus calculated by multiplying the number of workdays lost because of absenteeism by the relative ratio of days lost as a result of presenteeism versus the number of days lost because of absenteeism using the formula shown below:

$$D_{\mathrm{pr}}=\mathrm{RR}\times D_{\mathrm{ab}},$$

where *D*_*pr*_ is the number of equivalent days of presenteeism, *RR* is the relative ratio of days lost due to presenteeism versus the number of days lost due to absenteeism (i.e., 3.26), and *D*_*ab*_ is the average number of days of suspension from employment.

The estimated number of equivalent days of presenteeism was then combined with the number of days lost because of absenteeism. The cost of absenteeism and presenteeism associated with each disorder was then estimated by multiplying the number of patients, the total equivalent days of both absenteeism and presenteeism, and the average daily earning for each age range using the formula shown below:

$$C_{\mathrm{ab}}+C_{\mathrm{pr}}=P_{o}\times \mathrm{Pr}\times \left(D_{\mathrm{ab}}+D_{\mathrm{pr}}\right)\times W,$$

where *C*_*ab*_ *+ C*_*pr*_ is the cost of absenteeism and presenteeism, *P*_*o*_ is the population size, *Pr* is the 12-month prevalence of anxiety disorders, *D*_*ab*_ is the average number of days of suspension from employment, *D*_*pr*_ is the number of equivalent days of presenteeism, and *W* is the expected daily earning.

Finally, by combining the costs of each disorder, the total costs of absenteeism and presenteeism for anxiety disorders as a whole was estimated. During the morbidity cost calculation, a variety of uncertain parameters were used. To reflect the uncertainty of the results, a probabilistic sensitivity analysis (PSA) [21,22] was conducted to estimate the mean cost and its 95% confidential interval (CI). The details of this method are described in the ‘Uncertainty’ section below. All the parameters and their distributions that were included in the model to calculate absenteeism and presenteeism are shown in Tables 1, 2, and 3.

#### Unemployment cost

Regarding the unemployment cost, we estimated this cost for each sex and age group by multiplying the number of patients, the difference in the percentage of employment between the general population and the patients, and the average yearly earning using the following formula:

$$\mathrm{UC}=\mathrm{Po}\times \mathrm{Pr}\times \left(E_{\mathrm{gen}}–E_{\mathrm{anx}}\right)\times W,$$

where *UC* is the unemployment cost, *Po* is the population size, *Pr* is the point prevalence of anxiety disorders, *E*_*gen*_ is the employment rate of the general population, *E*_*anx*_ is the employment rate of patients with anxiety disorders, and *W* is the average yearly earning. The total unemployment cost was estimated by summating each category of sex-age-specific unemployment costs.

The number of patients in each sex and age group was calculated by multiplying the population [23] by the prevalence rate [6]. On the other hand, the employment rate for each sex and age group was calculated based on the *ASUKURI* research database [16] for patients with anxiety disorders and the Labour Force Survey [24] for the general population, We assumed that the employment rate of patients with anxiety disorders who do not receive treatment was the same as that of the general population. Thus, we estimated the employment rate of the patients with anxiety disorders to be as follows:

$$E_{\mathrm{anx}}=E_{\mathrm{anx}-\mathrm{tre}}\times R_{\mathrm{acc}}+\left(E_{\mathrm{anx}-\mathrm{ntre}}\times \left(1-R_{\mathrm{acc}}\right)\right),$$

where *E*_*anx*_ is the employment rate of the patients with anxiety disorders, *E*_*anx-tre*_ is the employment rate of the patients with anxiety disorders who receive treatment, *E*_*anx-ntre*_ is the employment rate of the patients with anxiety disorders who do not receive treatment, and *R*_*acc*_ is the rate of the patients with anxiety disorders who access treatment (i.e. 0.139 [S.E. 0.023] [6]).

When estimating the unemployment cost, we used various uncertainty parameters. To reflect the uncertainty of the result, we performed a PSA and calculated the mean cost and its SE. The PSA method is described in detail in the Sensitivity analysis section. The parameters inputted in the calculation of the unemployment cost are shown in Table 4.

**Table 4:** Parameters inputed for calculation of unemployment costs

**Male**						
Age	Employment rate (general population)*1	Distribution	Employment rate (patients with anxiety disorders)(S.E.)*2	Distribution*3	Expected yearly earinings (general population) (JPY:thousand)*4	Distribution
20-24	0.639	deterministic	0.435 (0.101)	beta	3,184	deterministic
25-29	0.885	deterministic	0.630 (0.070)	beta	4,029	deterministic
30-34	0.924	deterministic	0.717 (0.058)	beta	4,833	deterministic
35-39	0.934	deterministic	0.667 (0.059)	beta	5,672	deterministic
40-44	0.941	deterministic	0.733 (0.051)	beta	6,472	deterministic
45-49	0.941	deterministic	0.746 (0.056)	beta	6,894	deterministic
50-54	0.929	deterministic	0.759 (0.078)	beta	6,905	deterministic
55-59	0.892	deterministic	0.714 (0.075)	beta	6,382	deterministic
60-64	0.725	deterministic	0.379 (0.089)	beta	4,353	deterministic
65-69	0.478	deterministic	0.400 (0.096)	beta	3,632	deterministic
70-	0.202	deterministic	0.400 (0.096)	beta	4,028	deterministic
**Female**						
Age	Employment rate (general population)*1	Distribution	Employment rate (patients with anxiety disorders)(S.E.)*2	Distribution*3	Expected yearly earinings (general population) (JPY:thousand)*4	Distribution
20-24	0.648	deterministic	0.380 (0.068)	beta	2,815	deterministic
25-29	0.718	deterministic	0.548 (0.058)	beta	3,392	deterministic
30-34	0.617	deterministic	0.469 (0.062)	beta	3,618	deterministic
35-39	0.622	deterministic	0.435 (0.051)	beta	3,851	deterministic
40-44	0.687	deterministic	0.394 (0.060)	beta	3,973	deterministic
45-49	0.729	deterministic	0.333 (0.056)	beta	3,820	deterministic
50-54	0.698	deterministic	0.451 (0.069)	beta	3,732	deterministic
55-59	0.600	deterministic	0.311 (0.059)	beta	3,499	deterministic
60-64	0.425	deterministic	0.135 (0.047)	beta	2,872	deterministic
65-69	0.255	deterministic	0.075 (0.036)	beta	2,713	deterministic
70-	0.085	deterministic	0.075 (0.036)	beta	3,043	deterministic

*1 Derived from Labour force survey.

*2 Derived from ASUKURI reseach.

*3 Employment rate of the patients with anxiety disorders was assumed to follow Beta distribution. All other parateters were deterministic values.

*4 Derived from Basic Survey on Wage Structure.

#### Mortality cost

The definition of mortality cost was the net present value (NPV) of the expected lifetime earnings loss caused by suicides arising from anxiety disorders. The cost was calculated by multiplying the estimated number of suicides arising from anxiety disorders by the NPV of the expected lifetime earnings. The NPV was calculated using the formula shown below:

$$\mathrm{NP}V_{q}=\overset{l}{\underset{n=q}{\Sigma }}\frac{E_{n}W_{n}}{{\left(1+i\right)}^{n-q}}$$

where *q* is the age at the time of death by suicide, *n* is the age of the patient if they had survived, *l* is the life expectancy, *E*_*n*_ is the employment rate at the age of *n, W*_*n*_ is the average yearly earning at the age of *n*, and *i* is the discount rate.

The total number of suicides was obtained from the Statistics of Suicide of the National Police Agency [25]. The rate of anxiety disorders among the subjects who had committed suicide was obtained from Kaga’s data [26], published in 2009. The reason that this ratio was chosen was that although the sample size was relatively small (n = 76), a psychological autopsy had been conducted for 76 of the suicides, and the demographic data of the sample was representative of the data for all the suicide cases in Japan.

The expected lifetime earning was calculated based on the Basic Survey on Wage Structure [18] and the Labor Force Survey [24]. First, the average monthly wage of the general population of employees (regularly provided cash wages) was calculated for each sex and age group. Then, the expected lifetime earning of the general population was calculated. Second, the employment rate for each sex and age group was obtained from the Labor Force Survey [24], and the NPV of the expected lifetime earning was calculated by multiplying the employment rate by the expected lifetime earning of the employee. The mortality cost per suicide was regarded as the NPV of the expected lifetime earning of the general population from the age at death until the average life span (that is, if the person who committed suicide had lived and had reached the average life span).

The discount rate was set at 3%, as this figure has been used frequently in recent international research [27]. Similar to the estimation of the morbidity cost, uncertain parameters (such as the rate of patients with anxiety disorders among the suicides) were included in the calculation of the mortality cost, and we performed a PSA to calculate the average and SE of the mortality cost. The details of the PSA are described in the Sensitivity Analysis section. The parameters used to calculate the mortality cost are shown in Table 5.

**Table 5:** Parameters for calculation of mortality cost

**Male**						
age	the number of suicides *1	distribution	expected lifetime earning(JPY: thousand)* 2	distribution	the rate of suicides caused by anxiety disorders (S.E.) *3	distribution
20-29	2,373	deterministic	121,766	deterministic	0.149 (0.041)	beta
30-39	3,396	deterministic	119,104	deterministic	0.149 (0.041)	beta
40-49	3,852	deterministic	94,737	deterministic	0.149 (0.041)	beta
50-59	4,986	deterministic	52,633	deterministic	0.149 (0.041)	beta
60-	7,639	deterministic	11,489	deterministic	0.149 (0.041)	beta
unkown	204	deterministic	63,317	deterministic	0.149 (0.041)	beta
**Female**						
age	the number of suicides *1	distribution	expected lifetime earning(JPY: thousand)* 2	distribution	the rate of suicides caused by anxiety disorders (S.E.) *3	distribution
20-29	1,065	deterministic	57,818	deterministic	0.149 (0.041)	beta
30-39	1,454	deterministic	50,972	deterministic	0.149 (0.041)	beta
40-49	1,118	deterministic	38,805	deterministic	0.149 (0.041)	beta
50-59	1,377	deterministic	20,822	deterministic	0.149 (0.041)	beta
60-	4,154	deterministic	4,704	deterministic	0.149 (0.041)	beta
unkown	20	deterministic	24,791	deterministic	0.149 (0.041)	beta

*1 Derived from Statistics of Suicides in 2008.

*2 Derived from Basic Survey on Wage Structure.

*3 Derived from Kaga.

### Sensitivity analysis

The best available evidence was collected to estimate the cost of anxiety disorders. However, many of the parameters used for the estimations had constant uncertainties. Thus, while estimating the indirect costs, we performed a PSA [21,22] and calculated the mean and SE of the costs caused by anxiety disorders.

The probability distribution of each parameter of the indirect cost is shown in the references or was prescribed based on the SE calculated for that cost. The PSA was performed using 5,000 micro-simulations with the macro-function of Excel 2007. Each parameter in each simulation was decided based randomly on the probability distribution.

## Results

### Direct costs

The direct cost caused by anxiety disorders in Japan in 2008 was estimated to be JPY49.7 billion (USD 426 million). The medical care cost was JPY 49.4 billion (USD 423 million), while the involuntary admission cost was JPY 19.0 million (USD 163,000), the cost of the Medical Care and Supervision Act in Japan was JPY 27.0 million (USD 231,000), and the social service cost was JPY 244 million (USD 2.09 million). The details are shown in Table 6.

**Table 6:** Direct cost

**(JPY: million)**			
	**Inpatient**	**Outpatient**	**Total**
Health care cost	**-**	**-**	49,442
Health care cost under health insurance schema	8,064	41,332	49,396
Treatment cost	7,657	26,114	33,771
Medication cost	407	15,218	15,625
Involantary admission cost The medical care and	19	0	19
supervision act cost	**-**	**-**	27
Social service cost	**-**	**-**	244
Total			49,686

### Indirect costs

#### Morbidity cost

##### Absenteeism and presenteeism

The average number of days and its SE for suspension from employment depending on each disorder were as follows: agoraphobia, 7.3 (6.14); SAD, 7.3 (6.14); specific phobia, 1.3 (0.84); panic disorder, 7.3 (6.14); and GAD, 8.7 (2.88) (Table 2) (days lost caused by agoraphobia and panic disorder were assumed to be the same as that of SAD because of the lack of available data). As previously described, a literature review for the ratio of presenteeism/absenteeism identified only one article. The result was a ratio of 3.26. A PSA was conducted using the parameters shown in Tables 1, 2, and 3. As a result, the average number of days of suspension from employment resulting from the absenteeism and presenteeism of patients with anxiety disorders was estimated (Table 2). The results of the PSA showed that the productivity loss from absenteeism and presenteeism for patients with anxiety disorders was estimated as JPY 1.38 trillion (USD 11.8 billion) (Table 7).

**Table 7:** Indirect cost

**(JPY: million)**		
	**mean**	**S.E.**
Morbidity cost	2,099,089	6,950
Absenteeism and presenteeism	1,381,347	6,465
Unemployment cost	717,743	2,070
Mortality cost	244,395	944
Total indirect cost	2,343,484	7,008

#### Unemployment cost

The employment rates of the general population and of subjects with anxiety disorders and the expected yearly earnings are shown in Table 3. By multiplying the number of patients, the difference in the percentage of employment between the general population and patients, and the average yearly earning, the lost employment costs were estimated, since the 12-month prevalence rate and the employment rate of the patients with anxiety disorders used in the estimation contained some uncertainty. Therefore, to reflect this uncertainty, a PSA was implemented. The results revealed that the mean unemployment cost related to anxiety disorders was JPY 718 billion (SE: JPY 2.07 billion) (USD 6.15 billion [SE: USD 17.7 million]) (Table 7).

#### Mortality cost

The total number of suicides in Japan in 2008 was 31,638. The mortality cost was estimated by multiplying the rate of patients with anxiety disorders among the suicides from Kaga’s data [26] by the number of suicides for each sex and age group, and then multiplying this figure by the expected lifetime earning. The rate of patients with anxiety disorders among all suicides contained some uncertainty. Therefore, to reflect this uncertainty in the results, a PSA was implemented, and the mean mortality cost and its SE were calculated. The results revealed that the mean mortality cost of anxiety disorders was JPY 244 billion (SE: JPY 944 million) (USD 2.09 billion [SE: USD 8.08 million]) (Table 7).

## Discussion and conclusions

**Figure 1:**
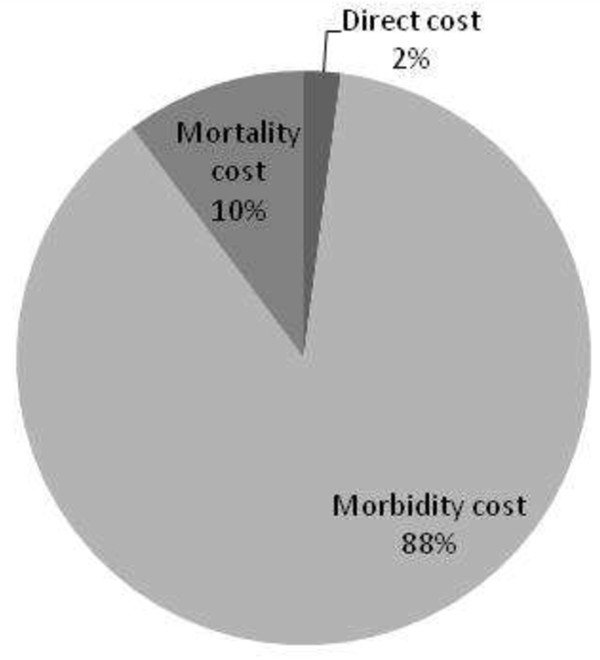
Proportion of each cost component.

From the results of this research, the total cost of anxiety disorders was estimated to be about JPY 2.39 trillion (USD 20.5 billion); the direct cost was about JPY 49.7 billion (USD 426 million), while the indirect cost was about JPY 2.34 trillion (USD 20.0 billion) (Table 8). Thus, indirect costs accounted for the major part of the total cost (Figure 1). In analyzing the costs of illness, it is difficult to conduct a direct comparison with other studies, as the cost categories included in the estimation and/or the estimation method differ among studies. Nonetheless, it became clear that in Japan, as in previous reports from overseas, the indirect costs constituted the major part of the total cost [7-9]. Furthermore, the estimated proportion of the direct cost in Japan was even lower than that in previous studies [7-9]. As one reason, we consider that the number of patients who actually consulted a medical institution is likely to be lower in Japan than in other countries. For example, in McCrone’s study [9], there were about 1,000,000 patients who were treated, whereas in the Patient survey [10], there were only about 500,000 adult patients. This difference probably affected the direct cost. However, the direct cost in Japan is still relatively low, even after adjustment for the lower number of patients. The reason for this is unclear, but the average treatment period of patients might be shorter in Japan. Regardless of this difference, one of the characteristic features of the costs of anxiety disorders is that the cost of indirect damage caused by the disease (indirect cost) is larger than the cost of treatment of the disease itself (direct cost).

**Table 8:** Total cost of anxiety disorders

	**Point estimate or Mean**		**S.E.**	
	**(JPY: million)**	**(USD: million)**	**(JPY: million)**	**(USD: million)**
**Direct cost**	**49,686**	**425**	**-**	**-**
Health care cost	49,442	423	-	-
Health care cost under health insurance schema	49,396	423	-	-
Involuntary admission cost	19	0.16	-	-
The medical care and supervision act cost	27	0.23	-	-
Social service cost	244	2.09	-	-
**Indirect cost**	**2,343,484**	**20,064**	**7,008**	**60.00**
Morbidity cost	2,099,089	17,972	6,950	60
Absenteeism and Presenteeism	1,381,347	11,827	6,465	55
Unemployment cost	717,743	6,145	2,070	18
Mortality cost	244,395	2,092	944	8.08
**Total cost**	**2,393,170**	**20,489**	**7,008**	**60.00**

On the other hand, what is the status of other mental disorders? Previous studies [3,28] have estimated the costs of schizophrenia and depression, respectively, using the same methodology as that used in the present study. As already implied by Sado et al. [3], a comparison of the results of the present study with those from previous studies indicates that for all 3 of these mental disorders, the indirect cost accounted for the greater part of the total cost (73% for schizophrenia; 91% for depression; and 98% for anxiety disorders).

Furthermore, when components of the indirect cost were examined, the costs caused by absenteeism and presenteeism constituted the largest component, accounting for about 59% of the whole. The unemployment cost was about 31%, and the mortality cost was about 10%. Similarly, in regard to the indirect cost component for other mental disorders, in the case of depression, the cost for absenteeism and presenteeism was about 53%, the unemployment cost was about 17%, and the mortality cost was about 30%. For schizophrenia, the unemployment cost was about 92% and the mortality cost was about 8%; absenteeism and presenteeism were not included as cost components in that estimation. However, as already discussed by Sado et al. [3], considering the low employment rate and the low prevalence rate of patients with schizophrenia, compared with those of patients with depression or anxiety disorders (both approximately one third of the employment rate and the prevalence rate of depression) [3,28], even if the costs of these components were estimated, it would not have had a large influence on the proportion of the indirect cost.

Based on these findings, when the cost caused by anxiety disorders is compared with that caused by other mental disorders, the decline in productivity as a result of absenteeism and presenteeism is the greatest for anxiety disorders. This is another unique feature of the cost of anxiety disorders.

According to the report of the WMH-J [6], the medical examination rate for anxiety disorders was about 14%, and this low medical examination rate may be another reason for the increased indirect cost. Therefore, the rate of medical care accessibility must be increased to provide proper medical treatment to individual patients and to reduce the costs caused by anxiety disorders, especially indirect costs.

### Limitations

Anxiety disorders are often considered to coexist with other mental disorders; however, we could not estimate the influence of this possibility of a dual diagnosis on the precision of the obtained data. This was one of the study limitations. Concerning the morbidity cost, this parameter was estimated using prevalence data from the WMH-J [6], and while the prevalence rate for each anxiety disorder was clear, the total prevalence rate was uncertain. Therefore, we estimated the total cost of anxiety disorders by adding the costs calculated for each anxiety disorder based on the prevalence rate of each anxiety disorder. Furthermore, concerning the mortality cost, the data for the rate of suicides among persons with each anxiety disorder was obtained from Kaga’s research [26]. However, in this research, suicides were counted as being caused by anxiety disorders even in patients with other coexisting disease. Thus, the results might have been overestimated. Furthermore, a human capital approach was adopted when evaluating the mortality cost. Compared with a friction approach [29], this method is likely to overestimate the impact.

Another limitation is the direct cost estimation. We had to assume that the average healthcare cost for anxiety disorders was the same as that for all F4 group disorders because of the lack of data regarding costs specific to anxiety disorders. However, this assumption might have caused an overestimation or an underestimation of healthcare costs, since the F4 group of disorders includes not only anxiety disorders but also other disorders, such as somatoform disorders, dissociative disorders, post-traumatic stress disorders, and so on, and service use by individuals with these disorders might differ from those with anxiety disorders.

Finally, the cost caused by all anxiety disorders was estimated in this study, even though it was revealed that the prevalence rate or the number of days of suspension from business differed among the various anxiety disorders. Thus, more precise suggestions for reducing indirect costs could likely be made if each anxiety disorder were to be considered in greater detail. This may be an important subject of study in the future.

## Competing interests

All the authors declare that they have no competing interests.

## Authors’ contributions

Mitsuhiro Sado (MS) drafted the protocol under the supervision of Ataru Inagaki (AI), Masaru Mimura (MM), and Kimio Yoshimura (KY). MS conducted the analysis. MS, Sayuri Takechi, Akihiro Koreki and Daisuke Fujisawa wrote the first draft of the manuscript. MM and KY commented upon it and provided feedback. All the authors have read and approved the final manuscript.

## Pre-publication history

The pre-publication history for this paper can be accessed here:

http://www.biomedcentral.com/1471-244X/13/338/prepub
